# Association of oral hygiene practices with the outcome of untreated dental caries and its clinical consequences in pre‐ and primary school children: A cross‐sectional study in a northern province of Saudi Arabia

**DOI:** 10.1002/cre2.438

**Published:** 2021-06-02

**Authors:** Ravi Kumar Gudipaneni, Santosh R. Patil, Ali A. Assiry, Mohmed Isaqali Karobari, Vinod Bandela, Kiran Kumar Metta, Redha Almuhanna

**Affiliations:** ^1^ Department of Preventive Dentistry, Pediatric Dentistry Division, College of Dentistry Jouf University Sakaka Al Jouf Saudi Arabia; ^2^ Department of Oral Medicine and Radiology New Horizon Dental College and Research Institute Bilaspur Chhattisgarh India; ^3^ Preventive Dental Science Department, Faculty of Dentistry Najran University Najran Saudi Arabia; ^4^ Conservative Dentistry Unit, School of Dental Sciences Universiti Sains Malaysia, Health Campus Kubang Kerian Kelantan Malaysia; ^5^ Fixed Division, Department of Prosthetic Dental Sciences, College of Dentistry Jouf University Sakaka Saudi Arabia; ^6^ Department of Conservative Dental Sciences Ibn Sina National College for Medical Studies Jeddah Saudi Arabia; ^7^ Saudi Ministry of Health Riyadh Saudi Arabia

**Keywords:** dental caries, dmft index, oral hygiene practice, pufa index, untreated dental caries

## Abstract

**Objectives:**

To assess the association amongst oral hygiene practices, untreated dental caries (UDC) and clinical consequences of UDC in pre‐ and primary school children aged 3–5 and 6–7 years.

**Materials and methods:**

A total of 250 subjects were recruited. The demographic and oral hygiene data were collected using a closed‐ended questionnaire. The UDC was measured using the ‘d/D' component of the decayed, missing, filled teeth (dmft/DMFT) index, and its clinical consequences were recorded using the ‘p/P' component of the pulpal involvement, ulceration, fistula and abscess (pufa/PUFA) index. The data were analyzed by multiple logistic regression.

**Results:**

Overall, 94.2% and 56.5% of the participants had one or more UDC and pulp involvement (*p* ≥ 1), respectively in 3–5‐year‐old age group. In the 6‐7‐year‐ age group the prevalence of UDC was 26.7% and the pulp involvement was 11.6%. Children who brushed with their fingers were 4.7 times more likely to have UDC (crude odds ratio [COR] = 4.71; 95% CI: 1.21–18.40). Twice‐daily brushing resulted in a 39% (*p* = 0.732) lower likelihood of having UDC compared with once‐daily brushing (COR = 0.61; 95% CI: 0.04, 10.09). Children with irregular brushing frequency were 3.2 times more likely to have pulpal involvement (COR = 3.21; 95% CI: 1.74–5.93).

**Conclusion:**

Finger brushing, irregular frequency of brushing and lack of parental supervision whilst brushing were associated with UDC and its consequences.

## 1 INTRODUCTION

Early childhood caries (ECC) is defined as the presence of one or more decayed (non‐cavitated or cavitated lesions), missing or filled surfaces of any primary tooth in children less than 72 months old (American Academy of Pediatric Dentistry, 2016). Globally, 621 million children have suffered from ECC, which poses a significant threat to children's oral health due to its consequences: chronic pain, odontogenic infections, sleep disturbances and tooth loss (Kassebaum et al., 2015). From 1990 to 2017, the Global Trends in Burden of Disease study showed that 2.3 billion children worldwide had untreated dental caries (UDC) in permanent teeth and 532 million children had UDC in primary teeth (Kassebaum et al., 2015). The highest prevalence rates of UDC are reported in Asia and Africa, where it affects 36% to 85% and 38% to 45% of children aged <6 years, respectively (Phantumvanit et al., 2018). In developing countries, more than 90% of dental caries in preschool children have not yet been treated (Anil & Anand, 2017). In the Kingdom of Saudi Arabia (KSA), the prevalence of dental caries in children is approximately 80%, demonstrating that the WHO's future goals for oral care have not achieved in the KSA (Alhabdan et al., 2018).

Dental caries is a sugar‐ and oral‐biofilm‐related disease due to the fermentation of carbohydrates by acidogenic microorganisms in the oral cavity. The presence of plaque deposits on the surface of the teeth due to ineffective or inadequate oral hygiene practices (OHP) promotes the onset of the caries process. Various risk factors are associated with the interaction amongst the susceptible host, agent and environment. The backbone of dental caries prevention is the removal of the oral biofilm with a toothbrush (Van Der Weijden & Slot, 2011). OHP may not be uniform across the globe due to the great diversity of cultures, beliefs and practices (Quadri et al., 2018). In addition, the factors that influence the effects of OHP on preschool children and the prevalence and severity of dental caries vary from region to region and from country to country. The socioeconomic status of the family, the knowledge and motivation of the parents and access to dental care play an important role in dental caries prevention (Anil & Anand, 2017). Furthermore, fluoride has played a critical role in the prevention of dental caries on a individual and community level. One of the most important public health strategies for reducing dental caries has been the widespread use of fluoride toothpastes (Toumba et al., 2019; Walsh et al., 2019).

Poor OHP is associated with the occurrence of dental caries (Oliveira et al., 2008). In addition to studying the OHP associated with tooth decay, the clinical consequences of UDC associated with poor OHP should be investigated. The northern province of the KSA has a high prevalence of UDC and its clinical consequences in preschool children (Gudipaneni, 2019; Gudipaneni et al., 2019). Therefore, the present study aims to evaluate the prevalence of UDC and its clinical consequences associated with OHP. Moreover, the association of OHP including oral hygiene methods, frequency of brushing and parental supervision whilst brushing with UDC and its clinical consequences amongst pre‐ and primary school children aged 3–5 and 6–7 years in the northern province of Al Jouf, KSA is explored. An overview of the OHP of these children will allow us to determine the potential effects of OHP on the outcome of UDC and its clinical consequences.

## 2 MATERIALS AND METHODS

### 2.1 Study population and sample size calculation

This study was conducted amongst pre‐ and primary school children aged 3–5 and 6–7 years who attended pediatric dental clinics in the College of dentistry, Jouf University, Al Jouf Province between August 2017 and March 2018. Al Jouf Province is located in the northern border region of the KSA, bordering Jordan. Systematic random sampling method was used to select the study participants. The minimum sample size required for the present study was estimated as 218 subjects with an absolute precision of 0.05, the anticipated proportion of dental caries 85%, significance level 0.05, and including 10% non‐participation. The participants were selected by using systematic random sampling method. Firstly, a random point was located with a fixed sampling interval. This sampling interval was obtained by dividing the estimated number of patients attending the pedodontics screening clinic with the estimated sample size for the present study. The total patients attended were 768 within the age group of 3–7 years, the sampling interval obtained was 3. Thus, we selected every third child attending the clinic every day until we reached the required sample size to make the study sample representative as possible to Al Jouf province. A total of 250 subjects were recruited and divided into two groups 3–5 years and 6–7 years. Amongst them, 138 subjects were in the 3–5‐year‐old age group, whilst 112 subjects were in the 6–7‐year‐old age group. The present study was conducted in accordance with the ethical guidelines of the Local Bioethics Committee, College of Dentistry, Jouf University, KSA. All measures followed in this study were in line with the Helsinki declarations. Only children whose guardians gave informed consent participated in our study. Children with special needs, hypoplastic defects or developmental abnormalities in dentition and those who did not want to participate in the study were not included.

### 2.2 Data collection

#### 2.2.1 Phase 1

**FIGURE 1:**
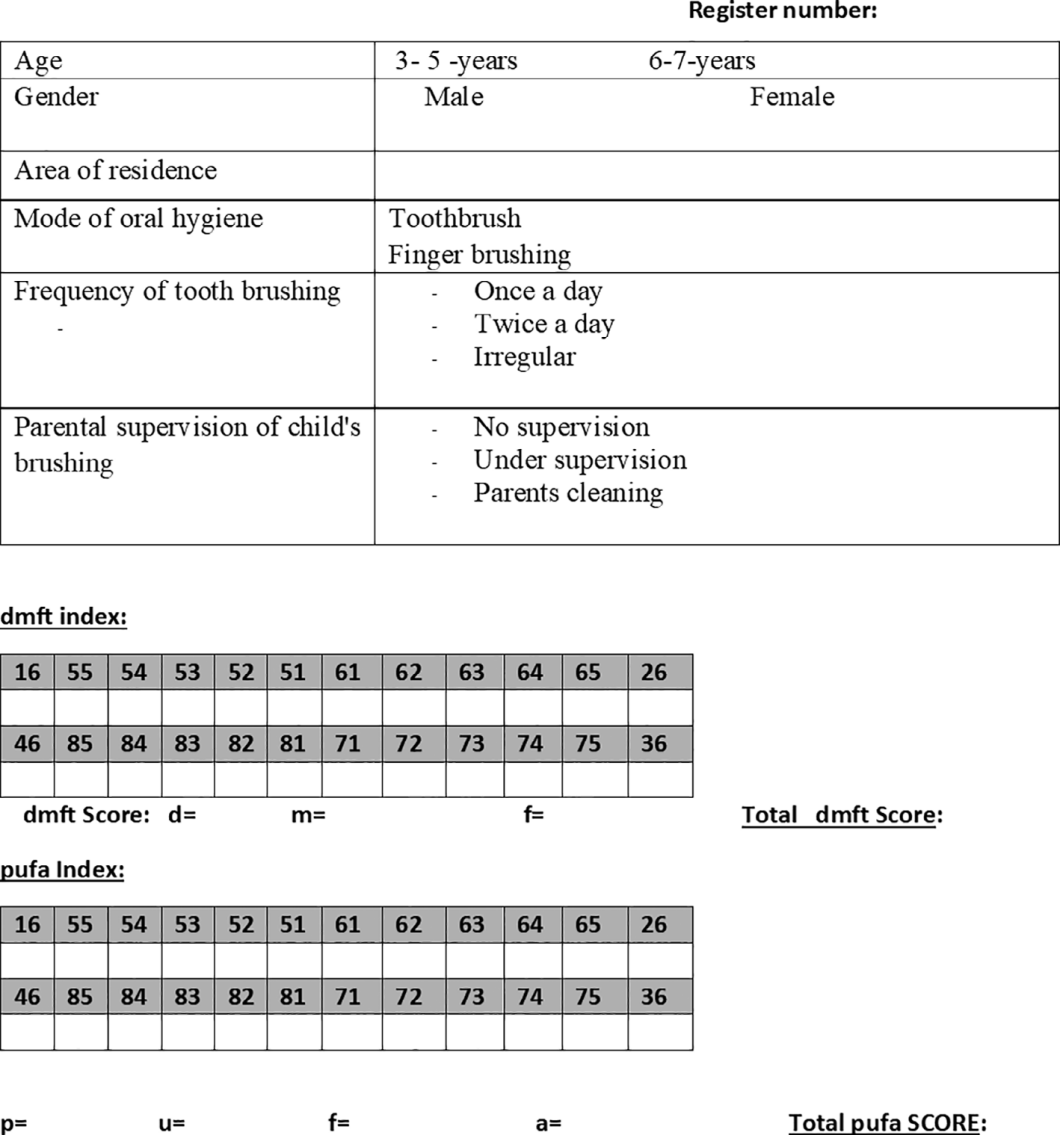
The questionnaire used in this study

The first phase involved the collection of participants' demographic and OHP data through a closed‐ended questionnaire filled by the parents/caretaker of the participant (Figure 1). Demographic information such as participant's age, gender and area of residence was collected. OHP data including mode of performing oral hygiene, frequency of brushing and parental supervision whilst brushing was obtained. Before the clinical examination, parents or caretaker of the participants were asked to fill the questionnaire during their initial visit to the pediatric dental clinics. The questionnaire was initially prepared in English and then translated into Arabic. Pre‐ and post‐tests were performed to calculate the reliability and validity (ICC = 0.78) of the questionnaire's items.

#### 2.2.2 Phase 2

The clinical examination was performed in the pediatric dental clinics in the College of dentistry, Jouf University, Al Jouf Province using artificial light, disposable mouth mirrors, wooden tongue blades and a WHO probe in the second phase. The UDC was measured using the ‘d/D' component of the dmft/DMFT (decayed, missing, filled teeth) index after drying the surface of teeth with cotton rolls and three‐way syringe. The consequences of UDC were recorded using the ‘p/P' component of the pufa/PUFA (visible pulpal involvement (P/p), ulceration caused by dislocated tooth fragments (U/u), fistula (F/f) and abscess (A/a)) index (Monse et al., 2010). No radiographs were taken to diagnose caries lesions in this study. Prior to clinical examination, investigators underwent training regarding the use of the dmft/DMFT and pufa/PUFA indices. Intra‐ and inter‐rater reliability was assessed using the kappa coefficient (κ) statistics.

### 2.3 Statistical analysis

An initial review of the data was performed to check for missing values and unusual observations. In the present study, descriptive statistics analysis was performed for prevalence estimates, and the association amongst gender, oral hygiene method, frequency of brushing, parental supervision whilst brushing, the presence of one or more UDC teeth (d ≥ 1) and the presence of one or more teeth with pulpal involvement (*p* ≥ 1) was analyzed using a chi‐square test. Independent *t*‐test was performed to find the mean difference in dmft/DMFT and pufa/PUFA scores between gender. The significance level was kept at 5% (*p* = 0.05).

In the multivariable analysis, simple logistic regression analysis was initially conducted to find the variables associated with the presence of UDC (d ≥ 1) and pulpal involvement (*p* ≥ 1). Variables with a *p*‐value <0.25 were considered essential and further used in multiple logistic regression to obtain the adjusted odds ratio. In multiple logistic regression, the forward LR and backward LR methods were used to include variables with a *p*‐value <0.05, and the final model using the enter method. The fitness of the final model was determined using the Hosmer–Lemeshow and receiver operating characteristic curve. All statistical analyses were performed using SPSS version 24 (IBM Corp., Armonk, NY, USA).

## 3 RESULTS

The reliability and reproducibility of each investigator was assessed by re‐examining 10% of the study population, and these children were excluded from further analysis. The intra‐rater kappa values for the two examiners (RKG and SRP) were 0.82 and 0.90 for the dmft and DMFT indices, respectively. The intra‐rater kappa value for the pufa/PUFA index was 0.74/0.81. The inter‐rater values for the dmft/DMFT and pufa/PUFA indices were 0.84/0.89 and 0.79/0.81, respectively.

Table 1 shows the descriptive statistics of the variables related to the population studied. The participants were divided into two groups according to their age, 3–5 and 6–7 years, to describe and compare the variables. The majority of the study population in both groups used toothbrushes to brush their teeth and had an irregular brushing frequency. Moreover, the majority of participants had no parental supervision whilst brushing their teeth, and no participant in the 6–7‐year‐old age group reported brushing their teeth under parental supervision. Only 19 (13.8%) children in 3–5‐year‐old age group reported being brushed by their parents.

**TABLE 1:** Descriptive analysis between study groups—expressed in N (%)

	Preschool (3–5 years)	6–7 years
Sample size	138	112
Biographic data		
Gender		
Male	83 (53.9)	71 (46.1)
Female	55 (57.3)	41 (42.7)
Oral hygiene practices		
Mode of oral hygiene		
Using toothbrush	109 (79)	102 (91.1)
Finger brushing	29 (21)	10 (8.9)
Frequency of brushing		
Once per day	39 (28.3)	24 (21.4)
Twice per day	10 (7.2)	29 (25.9)
Irregular	89 (64.5)	59 (52.7)
Parental supervision of child's brushing		
No supervision	111 (80.4)	99 (88.4)
Under supervision	8 (5.8)	0
Parents cleaning	19 (13.8)	13 (11.6)

Table 2 shows the mean dmft/DMFT and pufa/PUFA indices of both groups. The mean dmft score in the 3–5‐year‐old age group showed a significant difference in terms of gender of the participants (*p* = 0.046). Girls showed slightly higher mean dmft scores than boys in both age groups (*p* < 0.001). The mean PUFA score in the 6–7‐year‐old age group showed a significant difference in terms of gender of the participants (*p* = 0.018). However, no significant difference was noted in the mean pufa score of the 3–5‐year‐old age group. The d/D component of the dmft/DMFT index was higher compared with the p/P component of the pufa/PUFA index in both age groups.

**TABLE 2:** Descriptive and inferential analysis of clinical parameters

Parameter	Boys	Girls	*p* value
Clinical parameters—dmft score in primary dentition (expressed in Mean ± SD)			
d	4.11 ± 2.96	4.94 ± 3.07	**0.032** ^*^
m	0.46 ± 1.02	0.38 ± 0.96	0.485
ft	0.38 ± 0.83	0.41 ± 0.78	0.797
dmft score	4.96 ± 3.08	5.72 ± 2.92	**0.046** ^*^
Clinical parameters—DMFT score in permanent dentition (expressed in Mean ± SD)			
D	0.07 ± 0.41	0.70 ± 1.11	**<0.001** ^*^
M	0.01 ± 0.17	0.04 ± 0.26	0.446
FT	0.01 ± 0.12	0.07 ± 0.39	0.111
DMFT score	0.05 ± 0.30	0.71 ± 1.269	**<0.001** ^*^
Clinical parameters—pufa score in primary dentition (expressed in Mean ± SD)			
p	1.01 ± 1.46	0.92 ± 1.09	0.570
u	0.01 ± 0.08	0.06 ± 0.24	**0.013** ^*^
f	0.15 ± 0.36	0.06 ± 0.27	**0.032** ^*^
a	0.02 ± 0.14	0.05 ± 0.22	0.180
pufa score	1.20 ± 1.41	1.10 ± 1.12	0.525
Clinical parameters—PUFA score in permanent dentition (expressed in Mean ± SD)			
P	0.09 ± 0.43	0.22 ± 0.77	0.099
U	0	0	–
F	0	0	–
A	0	0	–
PUFA score	0.02 ± 0.19	0.15 ± 0.60	**0.018** ^*^

*Note*: Independent *t* test (test of significance) is applied at 95% Confidence interval (CI).

^*^ *p* value<0.05 was considered as statistically significant.

### 3.1 Prevalence of UDC and pulp involvement in the 3–5‐year‐old age group

Table 3 describes the occurrence of one or more UDC (d ≥ 1) and one or more teeth with pulp involvement (*p* ≥ 1) in the 3–5‐year‐old age group. Overall, 94.2% of the participants had one or more UDC. A significant difference in the prevalence of UDC (d ≥ 1) was observed in boys compared with girls (*p* = 0.035). A significantly high prevalence of UDC was observed in children using their fingers for brushing compared with those using a toothbrush (*p* < 0.001). A low prevalence of UDC was observed in children brushing twice daily compared with those brushing once daily (*p* < 0.001) and in children under parental supervision whilst brushing (*p* < 0.001). Furthermore, a high prevalence of UDC was observed in children with irregular brushing frequency and lack of parental supervision whilst brushing (*p* < 0.001).

**TABLE 3:** Descriptive analysis of untreated dental caries and pulp involvement amongst age groups—expressed in N (%)

	3–5 years				6–7 years			
Variables	Caries present d ≥ 1 *n* (%)	*p*‐value	Pulp involvement present p ≥ 1 *n* (%)	*p*‐value	Caries present D ≥ 1 *n* (%)	*p*‐value	Pulp involvement present P ≥ 1 *n* (%)	*p*‐value
Gender								
Male	77 (59.2)	0.035^*^	49 (62.8)	0.024^*^	19 (63.3)	0.144^*^	10 (76.9)	0.052^*^
Female	53 (40.8)		29 (37.2)		11 (36.7)		3 (23.1)	
Total	130 (94.2%)		78 (56.5%)		30 (26.7%)		13 (11.6%)	
Mode of oral hygiene								
Finger brushing	104 (80.0)	<0.001^*^	64 (82.1)	<0.001^*^	26 (86.7)	**<0**.001^*^	9 (69.2)	0.166^*^
Using toothbrush	26 (20.0)		14 (17.9)		4 (13.3)		4 (30.8)	
Frequency of brushing								
Once per day	38 (29.2)	<0.001^*^	18 (23.1)	<0.001^*^	9 (30.0)	0.003^*^	5 (38.5)	0.405^*^
Twice per day	9 (6.9)		5 (6.4)		3 (10.0)		0 (−)	
Irregular	83 (63.8)		55 (70.5)		18 (60.0)		8 (61.5)	
Parental supervision of child's brushing								
No supervision	104 (80.0)	<0.001^*^	62 (79.5)	<0.001^*^	30 (100)	–	13 (100)	–
Under supervision	8 (6.2)		0 (−)		0 (−)		0 (−0)	
Parents cleaning	18 (13.8)		16 (20.5)		0 (−0)		0 (−0)	

*Note*: Chi‐square test (test of significance) at 95% Confidence interval (CI).

^*^ *p* value<0.05 was considered as statistically significant.

The overall prevalence of teeth with pulp involvement (*p* ≥ 1) in the 3–5‐year‐old age group was 56.5%. A significant difference in teeth with pulp involvement was observed in relation to gender, wherein boys had more teeth with pulp involvement than girls (*p* = 0.024). A significantly higher prevalence of teeth with pulp involvement was observed in children using their fingers for brushing than those using a toothbrush (*p* < 0.001). The prevalence of teeth with pulp involvement was higher in children with irregular frequency of brushing compared with those brushing once a day and twice a day (*p* < 0.001). No children were reported with *p* ≥ 1 in children brushing their teeth under parental supervision (*p* < 0.001).

### 3.2 Prevalence of UDC and pulp involvement in the 6–7‐year‐old age group

Table 3 shows the occurrence of one or more UDC (D ≥ 1) and one or more teeth with pulp involvement (P ≥ 1) in the 6–7‐year‐old age group. Overall, the prevalence of UDC was 26.7%. A significantly higher prevalence of UDC was observed in children using their fingers for brushing compared with those using a toothbrush (*p* < 0.001). A high prevalence of UDC was observed in children with irregular frequency of brushing, whereas a low prevalence of UDC was reported in children who brushed once daily followed by those who brushed twice daily (*p* = 0.003). All participants in the 6–7‐year‐old age group reported no parental supervision whilst brushing. In terms of pulpal involvement (P ≥ 1), a significantly higher prevalence was observed in boys than in girls (*p* = 0.052). Children who used their fingers for brushing showed a higher prevalence of pulpal involvement teeth than those using a toothbrush. No observations of P ≥ 1 were reported in children who brushed their teeth twice daily and those with parental supervision whilst brushing.

### 3.3 Association between variables and the occurrence of UDC and pulp involvement

#### 3.3.1 Presence of UDC

**FIGURE 2:**
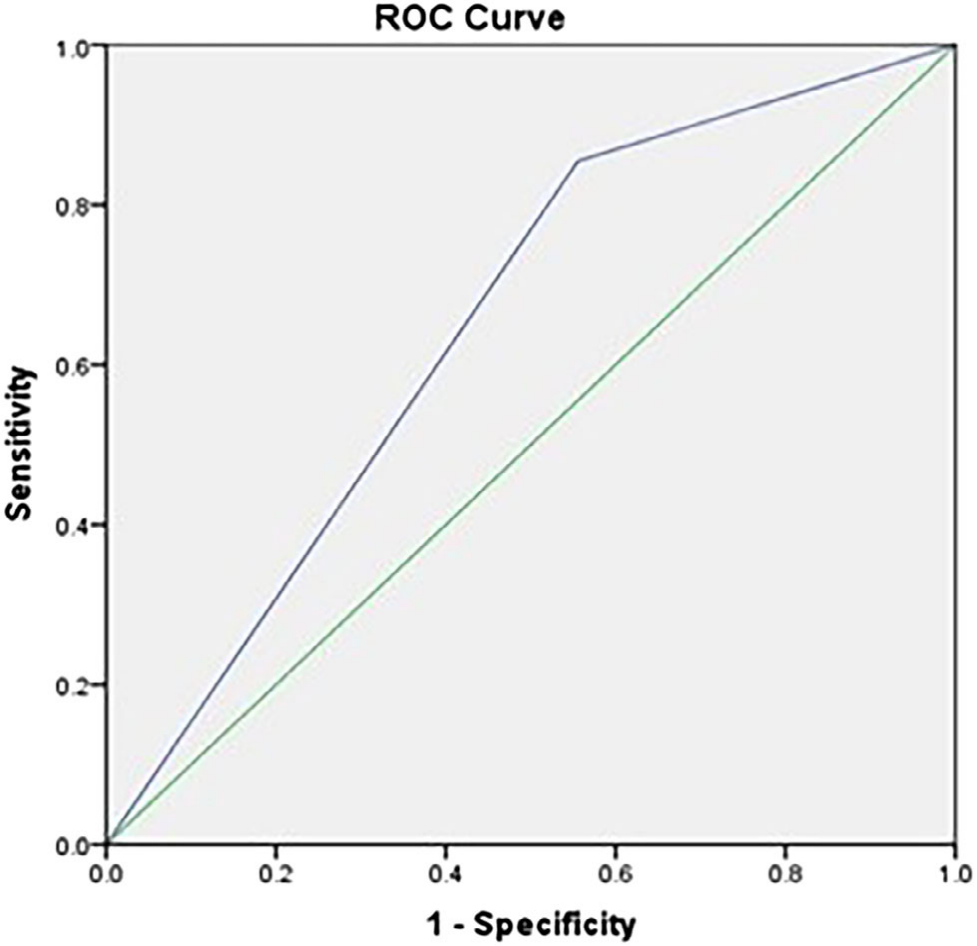
Model fitness for variables associated with occurrence of dental decay

Table 4 demonstrates the association between variables and the occurrence of ≥1 UDC and ≥ 1 pulp involvement in the study participants. The 6–7‐year‐old age group was more likely to have UDC 6.8 times higher than the 3–5‐year‐old age group (crude odds ratio [COR] = 6.83; 95% CI: 0.84–5.54). Girls were 26% more likely to have UDC than boys (COR = 1.26; 95% CI: 0.31–5.15). Children using their fingers for brushing were 4.7 times more likely to have UDC than those using a toothbrush (COR = 4.71; 95% CI: 1.21–18.40). Children who brushed their teeth twice a day were 39% less likely to have UDC than those who brushed once a day (COR = 0.61; 95% CI: 0.04–10.09). Age groups and oral hygiene mode were included in the multiple logistic regression analysis, and only oral hygiene mode remained significant (*p* = 0.026). The final model, only with oral hygiene mode, has an area under the curve (AUC) of 65% and Hosmer–Lemeshow *p*‐value of 0.200. Therefore, the final model has an adequate fit (Figure 2).

**TABLE 4:** The association between variables and presence of caries and pulp involvement amongst study participants

Variables	Caries present (decay ≥1)		Pulp involvement present ≥1	
	Multi variate regression		Multi variate regression	
	Odds ratio (95% CI)	*p* value	Odds ratio (95% CI)	*p* value
Age groups				
3–5 years	1	0.072	1	0.375
6–7 years	6.83 (0.84, 5.54)		0.80 (0.48, 1.32)	
Gender				
Male	1	0.751	1	0.063
Female	1.26 (0.31, 5.15)		1.64 (0.98, 2.75)	
Oral hygiene practices				
Mode of oral hygiene				
Using toothbrush	1		1	
Finger brushing	4.71 (1.21, 18.40)	0.026^*^	1.45 (0.73, 2.88)	0.286
Frequency of brushing				
Once per day	1		1	
Twice per day	0.61 (0.04, 10.09)	0.732	1.21 (0.53, 2.74)	0.648
Irregular	0.33 (0.04, 2.70)	0.298	3.21 (1.74, 5.93)	<0.001^*^

^*^ *p* value<0.05.

#### 3.3.2 Pulp involvement

**FIGURE 3:**
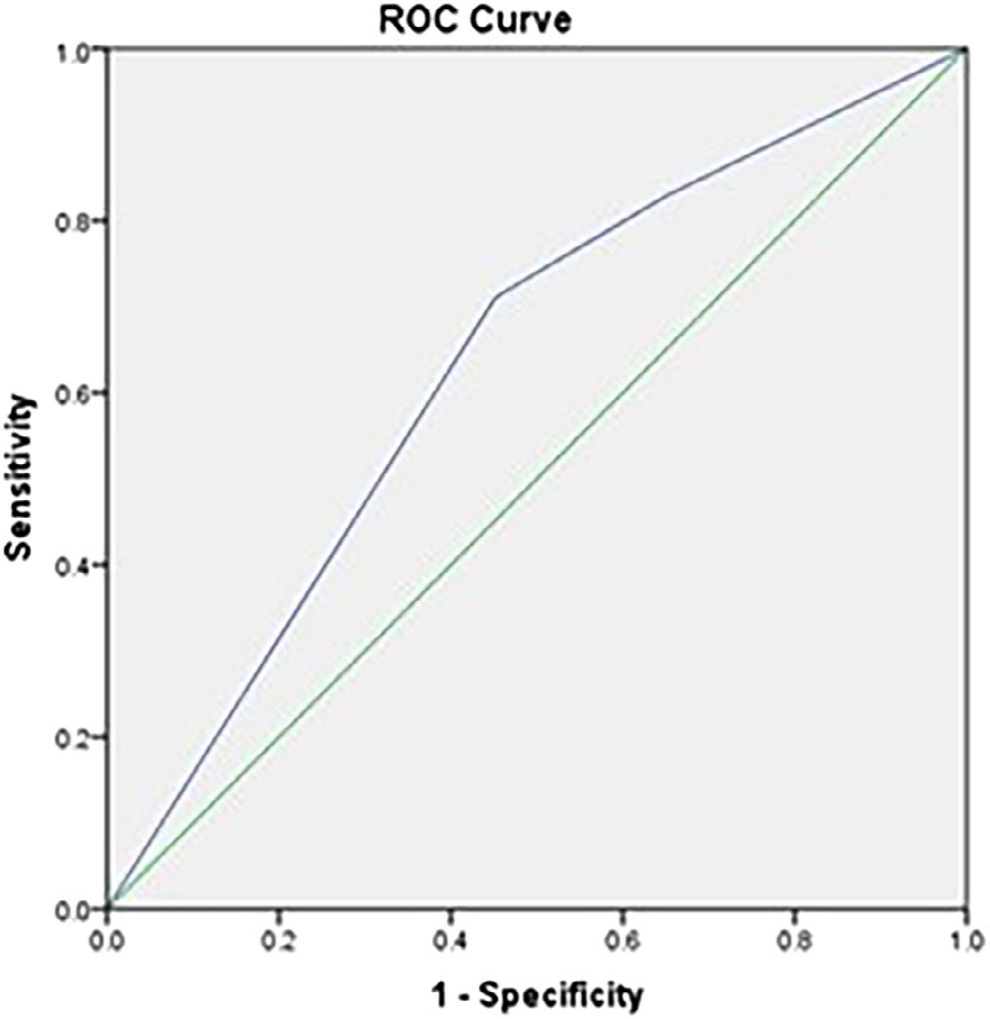
Model fitness for variables associated with occurrence of pulp involvement

The 6–7‐year‐old age group was 20% less likely to have pulp involvement than the 3–5‐year‐old age group (COR = 0.80; 95% CI: 0.48–1.32). Girls were 64% more likely to have pulp involvement than boys (COR = 1.64; 95% CI: 0.98–2.75). Children who used their fingers for brushing were 1.45 times more likely to have pulpal involvement than those using a toothbrush (COR = 1.45; 95% CI: 0.73–2.88). Children with irregular frequency of brushing were 3.2 times more likely to have pulp involvement than those who brushed once daily (COR = 3.21; 95% CI: 1.74–5.93). Gender and brushing frequency were included in the multiple logistic regression analysis, and only brushing frequency (irregular vs. once per day) remained significant (*p* < 0.001). The final model, only with brushing frequency, has an AUC of 63.3% and Hosmer–Leme show *p*‐value of 1.000. Therefore, the final model has an adequate fit (Figure 3).

## 4 DISCUSSION

The study aims to evaluate OHP as determinants of UDC and its clinical consequences amongst pre‐ and primary school children in the northern province of Al Jouf, KSA. Finger brushing, irregular frequency of brushing and lack of parental supervision whilst brushing were predominantly associated with UDC and its consequences. The present study provides evidence of the very high prevalence of UDC in the 3–5‐year‐old age group (94.2%) in the northern region of the KSA. However, a meta‐analysis in the KSA reported that the prevalence of dental caries in primary teeth is 5.38 (95% CI: 4.31–6.43) (Khan et al., 2013). In the present study, the 6–7‐year‐old age group had a higher risk of dental caries (OR = 6.83; 95% CI: 0.84–5.54) than the 3–5‐year‐old age group. A previous study found that the prevalence of caries and severity increase with age due to the increased frequency of consumption of sugary and carbonated beverages, modification of dietary habits and improper OHP amongst Saudi children (Farooqi et al., 2015). By contrast, surveys in England in 2012 (Davies et al., 2013) and Scotland in 2015–2016 (Hamilton et al., 2017) reported an approximately 28% reduction in caries in 5‐year‐old children.

A systematic review (Sarumathi et al., 2013) reported a significant association between the mode of oral hygiene and the prevalence of caries. In the present study, children who used their fingers for brushing were 1.89 times more likely to develop dental caries than those who used a toothbrush (OR = 1.89; 95% CI: 1.03–3.49). Because the fingers cannot effectively clean the fissures, the deep grooves and interproximal surface of the teeth become susceptible to caries (Sarumathi et al., 2013).

Toothbrushing is considered as a dynamic self‐care behavior, and a brushing frequency of two times per day has become a social norm, but the evidence for twice a day frequency of brushing is weak (Kumar et al., 2016). In our study, children who brushed twice a day had a significantly low prevalence of UDC and its clinical consequences. This finding is consistent with other studies that reported a significant relationship between dental decay and toothbrushing frequency amongst Saudi children (Alhabdan et al., 2018; Farsi et al., 2013). A high incidence and increment of dental caries were found in the primary teeth than the permanent teeth of children with irregular brushing frequency (OR = 1.39; 95% CI: 1.29–1.49) (Holmes, 2016). This is because the primary teeth are more susceptible to tooth decay than permanent teeth (Lynch, 2013). A meta‐analysis reported that people who do not brush their teeth once a day are 1.5 times more likely to have dental caries than children who brush their teeth regularly (OR = 1.56; 95% CI: 1.37–1.78) (Kumar et al., 2016).

In our study, the majority of the participants had irregular brushing frequency, which was associated with the high occurrence of UDC and its clinical consequences in both age groups. These findings are consistent with other studies reported from other regions of Saudi Arabia (Alhabdan et al., 2018; Farooqi et al., 2015). According to the Australian Dental Association (ADA, 2016), the frequency of toothbrushing should be twice a day for 2 min to obtain the maximum benefit (ADA, 2016). However, only a few studies reported a positive relationship with poor predictive accuracy (Turton et al., 2016). These findings showed that the quality of brushing the teeth is more important than brushing twice a day.

In our study, most children were not supervised by their parents when brushing. The most common barriers to parental behavior for brushing a child twice a day are the lack of adequate time and an uncooperative child behavior, stress, poor family organization and routine management (de Jong‐Lenters et al., 2019). A study reported that self‐efficacy, planning and action control are required for parents or caregivers to supervise children in brushing their teeth (Hamilton et al., 2017). Furthermore, supervised school toothbrushing programmes may reduce the increment of caries amongst the high‐risk population for dental caries (Clark et al., 2019). Schools often provide an opportunity for children to acquire the importance of regular OHP, prevention strategies and oral health education and to adopt long‐term skills to continue and achieve good oral health. A nationwide population study conducted in Scotland to assess the association between nursery toothbrushing programme and dental decay in five year olds reported an improvement in the dental health inequalities and a reduction in dental caries due to the introduction of the programme (Macpherson et al., 2013). However, a systematic review reported no strong evidence that supervised toothbrushing enhances the anti‐caries effect provided by fluoride toothpaste (Dos Santos et al., 2018).

In this study, the overall prevalence of caries amongst children in the 3–5‐ and 6–7‐year‐old age groups was 94.2% and 26.7%, whereas the prevalence of pulp involvement in both groups was 56.5% and 11.6%, respectively. Almost half of the ‘decayed' component has advanced to odontogenic infections as shown by pufa/PUFA index in this study with the majority of pulp involvement component (‘p/P'). Finger brushing and irregular brushing frequency in both age groups were associated with a high prevalence of pulpal involvement. The prevalence of UDC consequences in the KSA is higher compared with that reported in other studies (Sudan et al., 2018). These findings indicate that oral health education needs to be promoted in children who fail to attend their first dental visits and who inadequately use dental services. In addition, early preventive measures for caries in younger children can reduce the burden of UDC. A study evaluated the influence of UDC and its consequences on the quality of life of Brazilian children which reported that the prevalence rate of UDC was 64.6%, and 17.9% showed clinical consequences of UDC (pufa/PUFA index >0; Mota‐Veloso et al., 2016). UDC and its clinical consequences showed a negative effect on the OHRQoL in school children (Mota‐Veloso et al., 2016). In addition, UDC can increase the risk of developmental defects of permanent successor teeth (Grund et al., 2015). The pufa/PUFA index describes four clinical stages of UDC and draft the background of dental caries. The data provided by the pufa/PUFA index, which is complementary to the dmft/DMFT index, will provide a basis towards developing policies for promoting oral healthcare.

In the present study, only three important OHP variables were investigated. However, we cannot ignore the modifying effect of other host‐related risk factors as determinants of ECC, including socio‐behavioral and environmental factors, especially child feeding practices. Moreover, the type of toothpaste (e.g., fluoride or non‐fluoride toothpaste) was not included as a variable considering that most children use fluoride toothpaste in the northern region as reported in a previous study (Gudipaneni, 2019). The duration of brushing, the design and quality of the toothbrush, the brushing technique and the age at which the child started brushing his/her teeth were not studied. However, parents may have difficulty in remembering this information. The risk profile of ECC in Saudi Arabia appears to be consistent with that in developed countries, where OHP and dietary habits were primarily important (Alhabdan et al., 2018). In addition, the importance of behavioral factors related to oral health and onset of caries associated with host‐related risk factors, which could vary considerably amongst residents due to the diversity of cultural and behavioral practices.

This study reported an association amongst oral health practices, UDC and its clinical consequences in pre‐ and primary school children. However, the concept of the current study design needs to be further supported by various prediction models to explore the obvious determinants, including possible confounders, in order to understand the risk factors associated with UDC and its clinical consequences. In Saudi Arabia, the lack of regular OHP, lack of parental support and proper oral health knowledge, frequent consumption of a cariogenic diet, child feeding practices, dietary habits and socioeconomic status are important risk factors associated with dental caries in elementary school children (Alhabdan et al., 2018; Farsi et al., 2013). However, because brushing the teeth is believed to prevent caries, further studies with various study designs need to confirm this relationship. The findings of the present study show a significant relationship between OHP and UDC and its clinical consequences. Oral healthcare professionals, parents and primary school teachers should be aware of the importance of OHP in the prevention of dental caries. Moreover, oral health policymakers should consider implementing nursery toothbrushing programmes at the preschool level and increase awareness and motivation towards the ‘first dental visit’. An early dental visit before 12 months of age contributes to a significant reduction in dental caries, especially amongst children who are prone to caries.

The present study has a few limitations. This study is a hospital‐based cross‐sectional study involving children attending pediatric dental clinics for their first visits. The study participants would be considered high‐risk children and would provide a skewed result compared with population studies. In addition, the WHO (1997) dental caries diagnostic method used in the present study would underestimate the prevalence of caries. A self‐administered questionnaire was used to collect data on self‐reported OHP, which may be subject to recall bias. Therefore, whether respondents' self‐reported brushing practices are related to their actual toothbrushing behavior remains unknown.

## 5 CONCLUSION

In the present study, a high prevalence of UDC and its clinical consequences was reported in children who brushed their teeth using their fingers, had irregular frequency of brushing and lacked parental supervision whilst brushing. Interventions should be aimed to encourage good oral health practices and provide anticipatory guidance to parents. Oral health promotion programmes should be designed to focus on areas of specific needs to address inadequate OHP on the oral health status of children.

## CONFLICT OF INTEREST

The authors have no conflict of interest to declare.

## FUNDING

This study did not receive any financial support.

## AUTHOR CONTRIBUTIONS

RKG, SRP conceived and planned the study. RKG, SRP carried out the study. RKG, AA, contributed to statistical analysis. RKG, SRP, MIK, contributed to the interpretation of the results. VB, KKM and RA took the lead in writing the manuscript by preparing the first draft. RKG, AA, MIK contributed editing and final draft. All authors provided critical review of the manuscript and helped shape the research, analysis, and manuscript.

## Data Availability

The data that support the findings of this study are available from the corresponding author upon reasonable request.
